# Voluntary exercise in mesothelioma: effects on tumour growth and treatment response in a murine model

**DOI:** 10.1186/s13104-020-05284-y

**Published:** 2020-09-15

**Authors:** Scott A. Fisher, Carolyn J. Peddle-McIntyre, Kimberley Burton, Robert U. Newton, Elly Marcq, Richard A. Lake, Anna K. Nowak

**Affiliations:** 1 National Centre for Asbestos Related Diseases (NCARD), Perth, Australia; 2 School of Biomedical Sciences, University of Western Australia, Perth, Australia; 3 Exercise Medicine Research Institute, Edith Cowan University, Perth, Australia; 4 School of Medical and Health Sciences, Edith Cowan University, Joondalup, Australia; 5 School of Human Movement and Nutrition Sciences, University of Queensland, St Lucia, QLD Australia; 6 Centre for Oncological Research, University of Antwerp, Antwerp, Belgium; 7 School of Medicine, University of Western Australia, Perth, Australia

**Keywords:** Asbestos, Mesothelioma, Exercise, MexTAg

## Abstract

**Objective:**

There is substantial evidence that exercise can safely reduce the risk of cancer and improve survival in different human cancer populations. Long latency periods associated with carcinogen–induced cancers like asbestos induced mesothelioma provide an opportunity to implement exercise as an intervention to delay or prevent disease development. However, there are limited studies investigating the ability of exercise to prevent or delay cancer, and exercise as a preventive strategy has never been assessed in models with a known carcinogen. We investigated the potential of voluntary exercise (VE) to delay development of asbestos related disease (ARD) in our well-characterised, asbestos induced MexTAg model of mesothelioma.

**Results:**

Asbestos exposed MexTAg mice were given continuous or delayed access to VE and ARD assessed over time. We found that the addition of VE did not affect ARD development in asbestos exposed MexTAg mice. However, non–asbestos exposed, aged matched control mice participated in significantly more VE behaviours, suggesting subclinical development of ARD after asbestos exposure had a greater impact on VE participation than age alone. These data highlight the importance of model choice and the potential limitation that some pre–clinical studies may not accurately represent the clinical paradigm, particularly in the context of prevention studies.

## Introduction

Mesothelioma is an asbestos induced cancer with poor prognosis. Treatment is usually palliative, with systemic therapy providing limited survival benefit [1, 2]. Despite mesothelioma having a long latent period (20-40 years) between asbestos exposure and diagnosis [3], there are currently no effective strategies to prevent mesothelioma onset after asbestos exposure. Carcinogen induced cancers progress through multiple genetic, epigenetic and immunological modification, culminating in clinically apparent disease [4]. It is logical to hypothesise that some intervention during disease latency might delay onset of, or even prevent mesothelioma development. Such studies in humans require large participant numbers and take many years. However, animal studies allow for pre-clinical testing of these hypotheses, and subsequent selection of effective treatments for clinical trials.

Strong epidemiological evidence indicates that physical activity is associated with a reduced risk of developing cancer [5]. Compared with being inactive, the highest levels of physical activity have been associated with a 25% reduction in incidence of breast and colorectal cancer [6, 7]. Collectively, epidemiological evidence suggests that physical activity has tumour-mitigating properties across many cancers. Exercise holds potential as an intervention to delay or prevent mesothelioma in asbestos exposed individuals. However, the ability of exercise to prevent or delay cancer has never been investigated in models with a known carcinogen.

Pre-clinical evidence demonstrates that voluntary exercise (VE) can suppress tumour initiation and growth in mouse models of breast, colorectal and lung cancer [8–13]. Exercise induced suppression of tumour initiation and growth may be mediated by effects on tumour metabolism and immune function. Inflammation is critical in cancer development and VE has been shown to attenuate the inflammatory response in mice following carcinogen exposure, promoting more efficient clearance of damaged cells [14]. Additionally, VE decreases tumour incidence and growth via regulation of key immune cells, including natural killer (NK) and effector lymphocytes [13]. Here we investigated the potential of VE to delay development of asbestos related disease (ARD) in our well–characterised, asbestos induced MexTAg mouse cancer model [15, 16].

## Main text

### Methods

#### Experimental design

Experiments were approved by the UWA AEC (RA/3/100/1514) in accordance with the Australian code for the use of animals in medical research [17]. The C57Bl/6 derived 266 MexTAg transgenic mouse model expressing the Simian Virus 40 (SV40) Large T antigen (TAg) under the control of the mesothelin promoter has been described [15, 16]. For all experiments MexTAg mice (28–30/group, M&F, 6–20 weeks) were randomly assigned to their experimental groups and housed 3 per cage (Thoren cage; 19.56 × 30.91 × 13.34 cm) under standard conditions with food (standard meat free mouse and rat diet. Specialty feeds, Perth W.A. Cereal grain based diet, 12 mm pellets, digestible energy 14 MJ/kg) and water provided ad libitum. Mice were euthanised via methoxyflurane anaesthesia immediately prior to cervical dislocation. The extended duration of experiments and overt nature of ARD prevented blinding of investigators to treatment groups.

#### MexTAg mice and asbestos instillation

MexTAg mice were exposed to crocidolite asbestos via two, 0.5 ml (3 mg) intraperitoneal (i.p.) injections per mouse at weeks 0 and 4, monitored at least twice weekly and euthanised when ARD became evident; commonly ascites induced abdominal distension. ARD development and mesothelioma incidence was confirmed on histology as previously described [15, 16].

#### Voluntary exercise (VE)

VE was assessed by placing an activated or locked low profile wireless running wheel (Med Associates Inc^®^, USA.) into each cage and data collected using the USB Interface Hub and computer running SOF 860 Wheel Manager software. Mice were acclimatised and trained on running wheels for 1 week prior to start of experiment. Actogram analysis was performed using SOF 861 Wheel Analysis software, while assessment of VE parameters such as time spent running and distance run was performed using Microsoft Excel. Data was collected 24/7.

#### Statistics

Comparisons between two individual, or three or more groups were performed using unpaired, nonparametric (Mann–Whitney) Student’s *t* test (with Holm-Sidak correction for multiple comparisons), or unpaired, ordinary one-way ANOVA with Dunnett’s correction for multiple comparison respectively, relative to first data point. Log rank (Mantel-Cox) analysis was performed on survival curves. Analyses were performed using Graph Pad Prism Software V8 (Graph Pad Software Inc., USA). We used R [18] with afex [19] and emmeans [20] packages to perform mixed model ANOVA to analyse differences in VE behaviour over time. p values ≤ 0.05 were considered significant.

### Results

#### Nocturnal peak in voluntary exercise

**Fig. 1:**
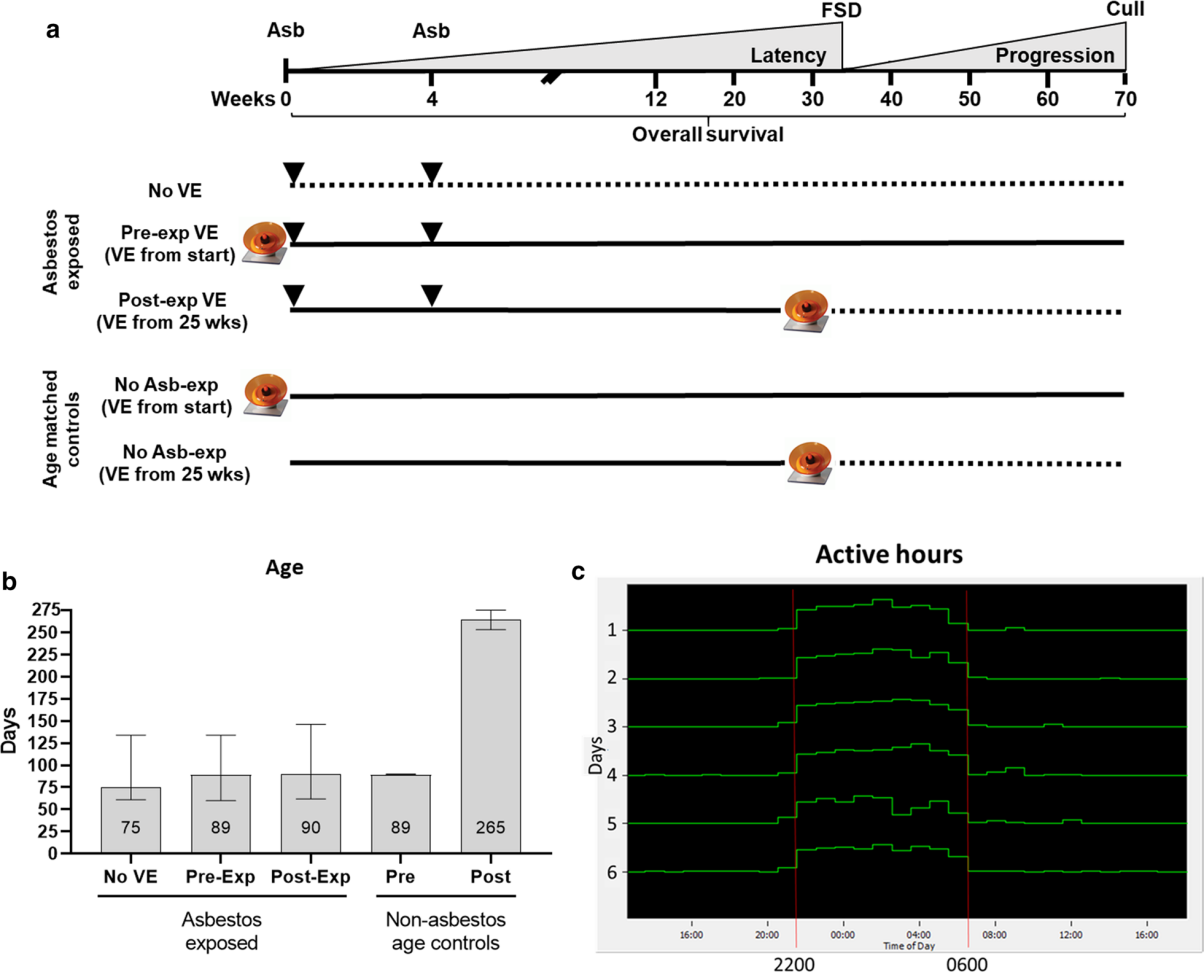
Peak VE activity occurs between 2200 and 0600 h. **a** Schematic of experimental design. MexTAg mice (n = 28-30/group, 3 mice per cage) were exposed to asbestos (6 mg total over 2 i.p. injections 4 week apart; black arrows) in the presence or absence of active running wheels (icons) as indicated. **b** Age distribution (median + range) between experimental and control groups. Age matched (Young: 90 days; Old (25 weeks) 265 days), non-asbestos exposed mice were used as controls to assess the impact of exercise over time in the absence of asbestos exposure. **c** Actogram depicting VE (active wheel running) over 6 consecutive days. Peak VE activity consistently occurred over an 8 h period between 2200 and 0600 h. Data shown are from a single cage from the Pre-Exp VE group and are representative of all VE groups. Data was collected 24/7 for 70 weeks and data between 2200 and 0600 h used for analysis

To assess the impact of voluntary exercise on ARD, asbestos exposed MexTAg mice (28–30/group) were randomly assigned to No VE or VE groups that either had continuous access to activated running wheels (Pre-exposure group (Pre–VE); starting 2 weeks before asbestos instillation), or from 25 weeks after asbestos instillation (Post exposure group (Post–VE), Fig. 1a). Age matched, non–asbestos exposed MexTAg mice served as controls to assess changes in exercise behaviour over time in the absence of asbestos (Fig. 1b). All mice were assessed for overall survival, while asbestos exposed mice were additionally assessed for disease latency; time from asbestos exposure to first signs of disease (FSD) and disease progression; time from FSD until cull.

Although voluntary exercise activity was collected continuously throughout the experiment, we first sought to identify the circadian pattern of VE. We observed high levels of running wheel activity between 2200 h and 0600 h, within any 24 h period for all VE groups (Fig. 1c). Based on these findings, all subsequent analysis of VE parameters was performed on data obtained within this period.

#### Voluntary exercise does not affect asbestos related disease development in MexTAg mice

**Fig. 2:**
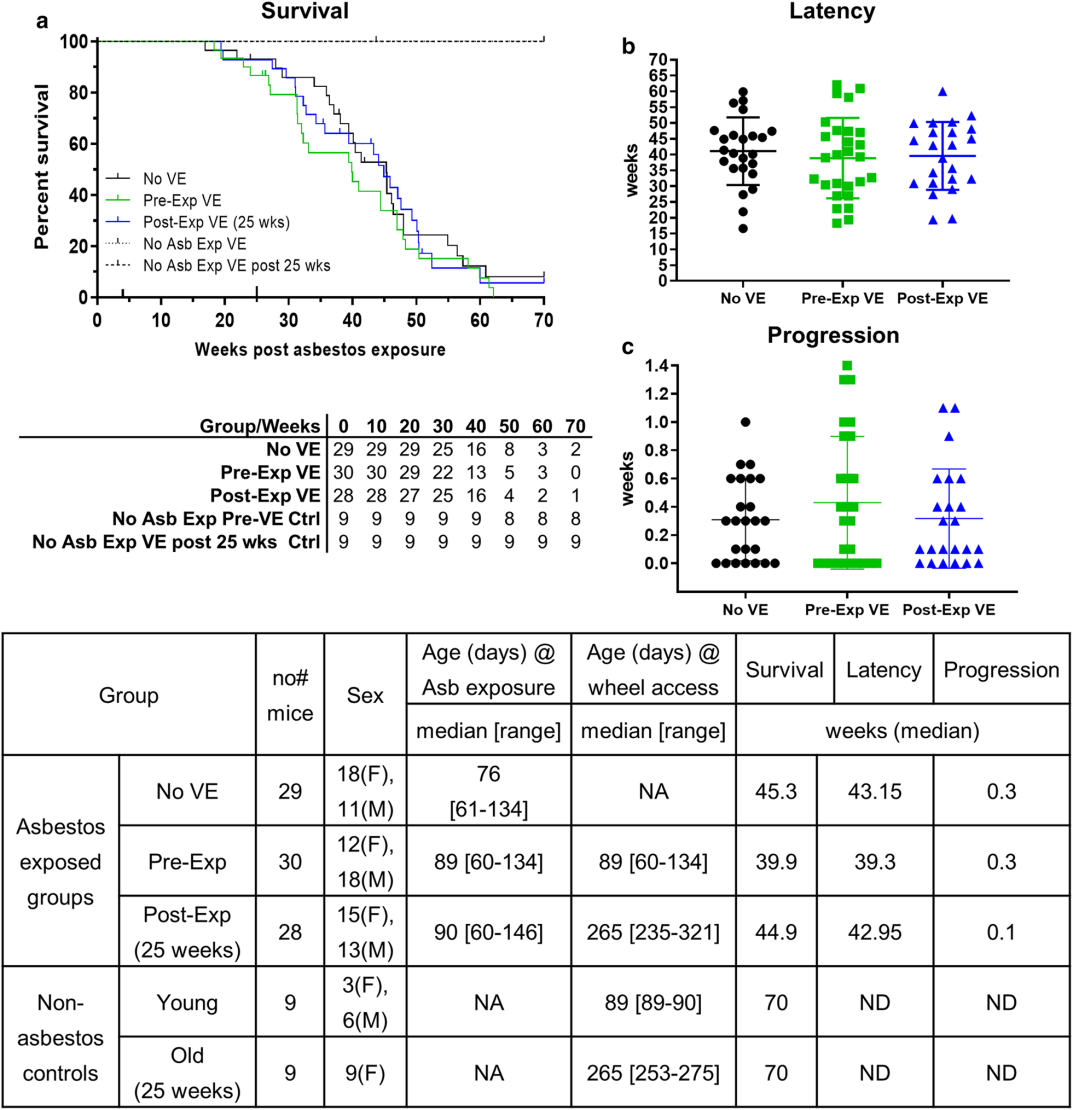
Voluntary exercise does not affect asbestos related disease in MexTAg mice. Crocidolite asbestos (6 mg total) was injected into MexTAg mice via two intraperitoneal injections four weeks apart. **a** Kaplan–Meier plot depicting survival over a 70 week period with the number of mice at risk shown in the corresponding table. **b** ARD Latency (time from asbestos exposure to first signs of disease) and **c** progression (time from first signs of disease until cull) in asbestos-exposed groups. Table define experimental design and cohort characteristics. Data are censored for asbestos related deaths and show mean ± SD. Log–rank (Mantel-Cox) analysis was used for survival. Kruskal–Wallis one-way ANOVA with Dunn’s test for multiple comparisons for all other analyses. p values ≤ 0.05 were considered significant. A single age-matched, non-asbestos exposed mouse was culled at week 44 in an incident unrelated to VE

No difference in median survival was observed between asbestos exposed mice given access to VE compared to the No VE control group (Pre-VE vs. No VE; 39.9 vs. 45.3 weeks, p = 0.2784; Post–VE vs. No VE; 44.9 vs. 45.3 weeks p = 0.7404), with all but 3 mice (2 from No VE and 1 from Post VE) succumbing to ARD by 70 weeks (Fig. 2a). No difference was observed in disease latency between asbestos exposed groups relative to No VE controls (Pre–VE 39.3 weeks (p = 0.72); Post–VE 42.9 weeks (p < 0.99 vs. No VE 43.1 weeks), or disease progression (Pre–VE 0.3 weeks (p < 0.99), Post–VE 0.1 weeks (p < 0.99) vs. No VE 0.3 weeks; Fig. 2b, c). In contrast, all age matched, non–asbestos exposed control mice survived to the 70 week experimental endpoint (Fig. 2a, dashed lines).

#### Asbestos exposure diminishes exercise participation

**Fig. 3:**
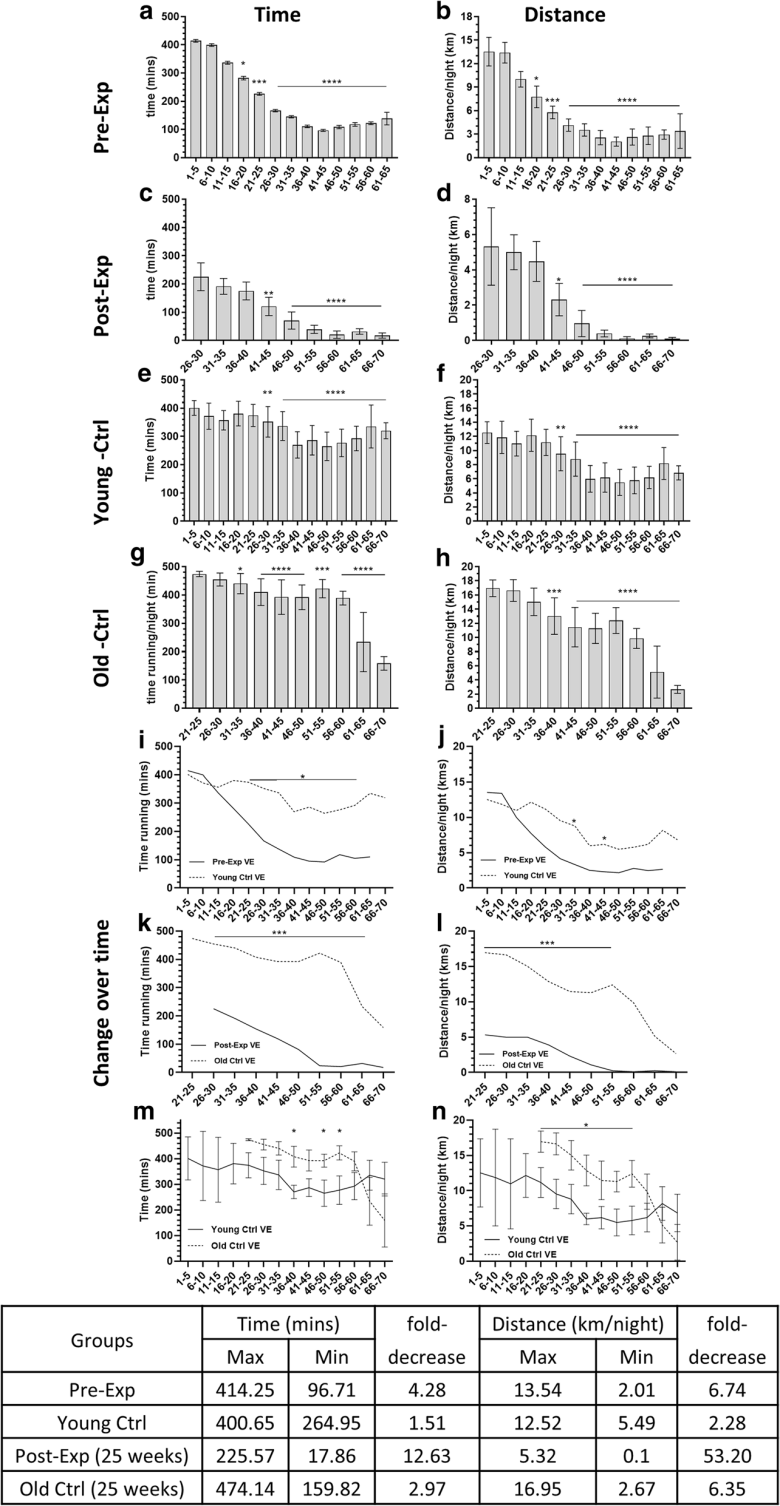
Diminished capacity for VE after asbestos-exposure. MexTAg mice given access to VE either 2-weeks before (Pre-Exp VE), or 25-weeks after (Post-Exp VE) asbestos exposure displayed a significant and sustained decrease in both the amount of time spent running/night (**a, c**) and total distance travelled/night (**b, d**) over a 70 week period. This was in contrast to age-matched, non-asbestos exposed MexTAg controls (**e–h**), which maintained significantly higher VE capacity over a similar time period. (**i-n**) Changes in VE over time between respective asbestos exposed and age-matched, non-asbestos exposed control groups (**i, j**: Pre-Exp VE vs Young Ctrl; **k, l**: Post-Exp VE vs Old Ctrl and **m, n** Young vs. Old controls). Table depicts fold-change over time. Data shown is mean ± SD. (**a–h**) Data analysed using a non-parametric Kruskal–Wallis one way ANOVA with Dunn’s test for multiple comparisons to start of VE. (**i–n**) Data analysed via non-parametric, mixed model ANOVA. * = p<0.05, ** = p<0.01, *** = p<0.001, **** = p<0.0001

We next assessed the time spent running per night and total distance run per night to determine whether VE participation changed over time. All asbestos exposed mice displayed a progressive decline in exercise (time and distance run per night) over the 70-week experimental duration. For Pre–VE and Post–VE groups, a significant decline for both time spent running per night and total distance run per night, was observed after 15 weeks of access to VE (Pre–VE running time/night: starting 16–20 weeks, p = 0.018 and distance/night: p = 0.028, sustained from 21 to 65 weeks, p < 0.0001; Post–VE time/night: starting 41–45 weeks (p = 0.002) and distance/night (p = 0.015), sustained from 46–70 weeks, both p < 0.0001; Fig. 3a–d). Whilst VE participation at 26–30 weeks was similar between Pre–VE and Post–VE groups, the Post–VE group showed a more profound and sustained reduction in exercise behaviours over time, with VE participation being almost negligible for mice surviving to 70 weeks (Fig. 3a, b vs. c, d).

To determine whether the decline in VE in asbestos exposed mice was affected by how old mice were when given access to VE, we repeated the experiment using non–asbestos exposed mice. Two groups, ‘Young’ or ‘Old’, were used as aged matched controls for Pre-VE (Young; 90 days at start of VE) and Post–VE (Old; 265 days at start of VE) groups respectively. Although a significant decrease in VE participation was observed within both control groups over time (p < 0.01), this decrease was not as marked in comparison to their respective asbestos-exposed groups (1.51 to 4.28-fold decrease vs 2.97 to 12.63 fold decrease non–asbestos vs asbestos exposed groups respectively). Magnitude of decrease in distance run per night was most pronounced in the Post–VE group (Fig. 3e–h).

This was further evident when we compared changes in VE over time between asbestos exposed VE groups and their respective control group, where both groups of asbestos exposed mice consistently spent significantly less time running, and ultimately travelled less distance per night, relative to their respective non–asbestos exposed control (Fig. 3i–n). Interestingly, VE participation in age matched, non-asbestos exposed controls in which VE was delayed (Old controls) was significantly higher (p < 0.05) relative to the continuous VE (Young) control group (Fig. 3m–n), suggesting that older mice might be more responsive to VE intervention. Taken together, these data indicate that asbestos exposed mice had reduced VE participation over time suggesting that the subclinical development of ARD after asbestos exposure had a greater impact on VE participation than age alone.

### Discussion

Here we employed our asbestos induced MexTAg mouse model to assess the impact of VE on ARD following asbestos exposure. In contrast to other studies in which VE delayed tumour growth [13], the addition of VE (continuous or delayed) did not affect ARD development in our model. Mesothelioma is unusual in that the carcinogen, asbestos, induces a similar disease in mice and humans. This is rare in cancer research and presents an ideal opportunity to apply small animal models to advance mesothelioma prevention and treatment. However, model choice may explain why VE did not have any significant impact on ARD in asbestos exposed MexTAg mice. Previous studies demonstrating significant VE associated reduction in tumour incidence and growth employed mouse models employing intravenously or subcutaneous tumours [8–13]. In these studies, VE enhanced tumour suppression was associated with increased expression of p53 and mediators of apoptosis [9], or mobilisation and redistribution of NK cells in an epinephrine and IL–6 dependent manner [13]. In contrast, the MexTAg model involves induction of ARD in situ following asbestos exposure, where TAg expression phenocopies p16 loss, effectively bypassing p53/p16 mediated cell cycle control [21]. Therefore, the inherent genetic modifications that drive the oncogenic potential in transgenic models like MexTAg might mask any benefit induced by supportive adjuvant therapies like VE.

While we did not observe differences in ARD, we did observe significant differences in VE participation between, and within asbestos exposed and non–exposed groups. In particular, VE participation was higher in age matched, non-asbestos controls in which VE was delayed (Old) relative to asbestos exposed delayed VE and the continuous (Young) VE control group; indicating that asbestos exposure, rather than age, had a greater impact on the observed reduction in VE participation over time. We also observed a decrease in exercise prior to clinical signs of disease development in asbestos exposed mice. It is important to consider these data in context of the clinical setting, in which mesothelioma patients are often elderly, have a sedentary lifestyle and present with high disease burden [22, 23]. Together, these data suggest that the impact of VE on asbestos induced ARD might be better observed and modelled using aged mice. Alternatively, our recent human data [24] demonstrated that a short (six–week) tailored resistance exercise training program was well tolerated and beneficial to mesothelioma patients. As data in this study also indicated a decrease in exercise preceded disease detection, this may suggest that patients presenting with mesothelioma might benefit from additional support and rehabilitation at diagnosis.

In conclusion, the addition of continuous or delayed VE did not significantly affect ARD development in asbestos exposed MexTAg mice. Our data is in contrast to previous studies and highlights the importance of choosing an appropriate model and rigorously evaluating model parameters. Preclinical transplant models might be useful for ‘proof of concept’ studies, but as seen here, may not be applicable across different tumour types and may not phenocopy in situ development of human cancer. Additionally, our study highlights that exercise alone may not be sufficient to counteract the oncogenic potential of strong carcinogens like asbestos. As such, some pre–clinical studies may not accurately represent the clinical paradigm, particularly in the context of prevention style studies, and therefore have limited translational impact.

## Limitations

Study-specific limitations include: 3 mice per cage–therefore, exercise data is not indicative of an individual mouse; Furthermore, similar ARD development across all groups might simply suggest that other modes of individualized exercise, rather than continuous VE used in this and other studies [8–13], might be more effective as adjunct supportive treatment for mesothelioma.

## Data Availability

The datasets used and/or analysed during the current study are available from the corresponding author on reasonable request.

## Abbreviations

Asb: Crocidolite asbestos
FSD: First signs of disease
i.p: Intraperitoneal
MexTAg: Asbestos induced mesothelioma mouse model
µm: Micrometre
mg: Milligram
SD: Standard deviation
SV40: Simian Virus 40
TAg: Large T antigen from SV40
VE: Voluntary exercise
NHMRC: National Health and Medical Research Council
